# Patient involvement at the managerial level: the effectiveness of a patient and family advisory council at a regional cancer centre in Sweden

**DOI:** 10.1186/s12913-021-07026-7

**Published:** 2021-09-25

**Authors:** Mio Fredriksson, Anton Modigh

**Affiliations:** Department of Public Health and Caring Sciences, Uppsala University, Box 564, 751 22 Uppsala, Sweden

**Keywords:** Patient and family advisory councils, Patient and public involvement, Cancer care, Cancer policy, Regional cancer centres, Service improvement

## Abstract

**Background:**

In cancer care as well as other types of treatment and care, little is known about the contribution of Patient and Family Advisory Councils (PFACs) operating at the *managerial level* in healthcare organizations. The aim of the study was to investigate the effectiveness of a Swedish PFAC operating at the managerial level at one of Sweden’s six regional cancer centres.

**Methods:**

This was a qualitative, single-case study based on interviews with PFAC participants and meeting minutes from PFAC meetings. These were analysed using a modified version of a framework developed by Abelson et al. to design and evaluate collective involvement processes in the healthcare sector: (i) representation; (ii) information; (iii) process or procedures and (iv) outcomes and decisions.

**Results:**

The descriptive representation was good regarding geographical location and cancer diagnosis. Information from the regional cancer centre was an important part of the meeting agenda. The procedures encouraged everyone to speak up, and the participants saw the representatives from the regional cancer centre as allies against the hospitals and regions, raising some questions about the PFAC's independence. Regarding outcomes, most participants did not know to what extent their work had led to any improvements in cancer care. However, they still regarded the council as effective, as issues the participants raised were listened to by the representatives from the regional cancer centre and ‘taken further’ in the healthcare organization.

**Conclusions:**

The participants were satisfied with being listened to, but they found it difficult to know whether their work had led to improvements, in part because they did not know enough about how the healthcare organization worked above the care-provision level. This was a hurdle to achieving change. The study suggests it is more difficult for patients and next of kin to participate at the managerial level, compared to the care-provision level in healthcare systems, where they could potentially influence important aspects of cancer care and policy, since it is at these levels strategic decisions about priorities in cancer care and service configurations are made. This indicates that there is a particular need for guidance and support for patient and public involvement to work successfully at this level, which may include relevant education and training in system knowledge.

## Background

Patient and public involvement (PPI) is increasingly common in health research and healthcare. In healthcare, PPI refers to the ‘active participation of citizens, users and carers and their representatives in the development of health care services and as partners in their own healthcare’ [1]. Two broad arguments are made for PPI. The first is a moral argument: those affected by, or paying for, research and health services should have a say in what and how they are conducted and delivered. The second is a consequentialist argument: it will improve the quality, efficiency and impact of research and healthcare [2].

PPI can be not only individual but also collective [3]. As such, involvement is not geared towards the individual level (i.e. the direct care setting, which is the most common level of involvement or engagement in cancer care [4]) but rather the clinic or organizational level and the policy level [5]. Involving patients in the design of healthcare systems and in quality improvement efforts has been described as a critical aspect of patient-centred care [6] and The European Cancer Patient Coalition (ECPC) encourages a strong partnership between researchers, cancer centres and patients, where patients can share their unique experiences and develop a more patient-centred approach to cancer care and research [7].

Fundamentally, there are three main ways to organize *collective involvement* in healthcare and in society as a whole [8]. The first is to invite a group of representative citizens, e.g. citizens’ juries, to voice their opinions, which may then be used to deliver recommendations applicable to policy and practice decision-making [9]. The second is to involve organized civil society groups, e.g. patient organizations or community groups. Research indicates great diversity among European countries as to the extent to which cancer patient organizations partake in policy decision-making and with what impact [10, 11]. The third is to invite a specific group of citizens to participate, the ones that are affected by a decision, e.g. people with cancer (which may not belong to an organization or similar).

Today, there is great variation in the methods and terminology used in different healthcare systems when it comes to involving particular groups, but there is a growing body of literature on Patient and Family Advisory Councils (PFACs). PFACs are groups of patients, family members and caregivers who meet regularly and serve as advisors in diverse healthcare settings, from individual clinics and hospitals to healthcare systems [6, 12]. Although some researchers conclude that PFACs can provide new insights that can assist healthcare providers in optimizing current methods [13] and change staff ‘culture’ or awareness [14], the impact of PFACs is uncertain, in particular regarding healthcare decisions at the population level [6, 14, 15]. Similarly, for PFACs in cancer care, it has been suggested that they seem to play an important role in the drive to make healthcare more patient-centred, but there is very little information about their efficacy [16]. In general, there is a lack of robust evidence for the impact of PPI in healthcare policy and service improvement [17–20].

As PFACs most often operate at the hospital level and include healthcare staff, the vast majority of studies on PFACs concern hospitals, i.e. the *operational* level within a healthcare organization. However, although unusual, this type of council may also operate at the *managerial* level, i.e. in parts of the healthcare organization that do not deliver healthcare but rather work to develop care and research. One such example is PFACs attached to the six regional cancer centres (RCCs) in Sweden. The RCCs have been part of Sweden’s national cancer strategy from 2009. The overarching goal of the RCCs is to work towards more equitable, accessible and patient-focused cancer care of high quality throughout the entire chain of care [21]. Although an overall assessment of the development of the RCCs concluded that one of the greatest merits is strengthened patient participation and patient-centring [22], it is uncertain what the outcomes of the RCC PFACs are, and if this is a type of involvement that leads to changes in cancer services or policy. In the literature, the outcomes or impacts of involvement are sometimes referred to as the ‘effectiveness’ of participatory activities [23]. The aim of the study was therefore to investigate the effectiveness of one of the Swedish RCC PFACs operating at the managerial level, with the aim to contributing to the development of cancer care. Evaluation has been suggested one of nine essential principles to optimize patient and public involvement [24].

### The RCC organization and patient representation

The responsibility for the funding and provision of healthcare in Sweden is placed with 21 self-governing regions, which work together in six larger clusters (cooperative regions). Each of the six cooperative regions, which are divided geographically, has a regional cancer centre (RCC) that works in cooperation with the regions within its cluster to develop and improve the quality of cancer care. Organizationally, the RCCs are linked to the regions in different ways because the regional forms of collaboration and decision-making differ, but they are all anchored in the regions’ leadership. The RCCs, which differ in size from about 25 to 60 employees, are managed by an operations manager that leads the work in a governing board with representatives from the regions within its cluster (health professionals and public officials).

The RCCs also work nationally to develop and improve the quality of cancer care. For example, the RCCs collaborate through a collaboration group where the RCC managers meet regularly with the national cancer care coordinator at the Swedish Association of Local Authorities and Regions (SALAR). The collaboration group advises the SALAR, the Ministry of Health and Social Affairs and the National Board of Health and Welfare on cancer care issues. For the period of 2020–2022, the RCCs focus their work on ten common development areas, three of which are prevention and early detection, staff competence, and patients and next of kin.

The RCCs have the fundamental task of supporting the patient’s position in healthcare. In practice, the involvement of patients and their next of kin takes place at several levels relating to the RCC’s work (Table 1). With the RCC as a main coordinating body in cancer care improvement, with a clear vision of involving patients and their next of kin, it becomes relevant to explore the effectiveness of involvement within the RCC setting. This article studies the councils for patients and their next of kin, which target the macro and meso levels within the healthcare organization (Table 1).

**Table 1:** Involvement of patients and their next of kin at the RCCs

	Description
Patient- and Family Advisory Councils (PFACs)	All six RCCs have a council for patients and their next of kin (referred to as PFACs in this article, *Patient- och närståenderåd* in Swedish). Participants must have experience with cancer themselves or as next of kin, and they are reimbursed for their time. The PFACs’ task is to monitor the perspective of patients and their next of kin in cancer care and the work carried out by the RCC. The intention is that the PFACs contribute to the development of cancer care, in particular to meet the needs of patients and their next of kin during the care process. The RCCs’ PFACs should focus on general issues that concern the group of patients and their next of kin, not diagnosis-specific issues or issues specific to individual patients. Furthermore, the PFACs have an advisory role in the RCC, and the participants should monitor and identify areas of improvement and present these to the RCC leadership and give feedback on proposals. The organisation and mode of operation of the RCCs’ six PFACs differ. According to a regional assignment description for the investigated PFAC, the PFAC members assign a chairperson and a vice chairperson amongst themselves. The chairperson is included in the RCC board, but has no decisional authority. The RCC supports the group by contributing with a coordinator, an RCC employee, who assists with administrative tasks such as taking minutes and distributing information in between meetings. The RCC operational manager participate at the PFAC meetings.
Care process groups	There are 15–30 *care process groups* at each RCC. These groups are tasked with leading and coordinating the development of care processes for the respective diagnosis in the cooperative region and consist of healthcare professionals who are clinically active in the current cancer diagnosis and 1–2 patient representatives. There are also patient representatives in the *national groups developing practice guidelines in cancer care*.
Local cancer councils	In most regions, there are also *local cancer councils* (LCCs), which coordinate the work between the RCC and the region. All regions in the collaborative region where the study was carried out have LCCs, and their chairpersons are also members of the RCC board. All the regions have collaborations with patient organizations. A majority of them have patient and next-of-kin representatives as members of the LCC; some regions have set up their own local council for patients and their next of kin. Many of the patient representatives at the local level are involved in the RCC’s PFAC.
National working group for patient collaboration	The working group is tasked with developing the forms of patient collaboration in cancer care. The group works to ensure that the patient and their next of kin have good and equal conditions for fulfilling their assignments at all RCCs. It consists of one employee per RCC and members from the six PFACs. However, this group is to be reorganized.

## Methods

### Design

The method used for this study was a qualitative, single-case study [25]. The study was conducted in one of the RCC PFACs, which have worked toward improving patient and next-of-kin representation to increase their influence.

### Data collection

Two types of data sources were used to address the purpose of the study: interviews with PFAC participants and meeting minutes from the PFAC meetings.

The interview study was conducted by first presenting the aim of the study to the PFAC participants at one of their meetings in early 2020. Thereafter, participants were invited to partake via email. They received written information about the study purpose and procedure and were informed that participation was voluntary. Those who did not answer received two reminders. At the end, ten of 15 potential PFAC members agreed to participate, among which the duration of participation, type of cancer and geographical location differed. They were interviewed via telephone since no face-to-face interviews were conducted during the COVID-19 pandemic. The interviews lasted between 24 and 57 min and were semi structured. The interview guide was developed using an analytical framework presented by Abelson et al. [22].

The document study was conducted by analysing meeting minutes accessible from the RCC web page. The minutes were written by the RCC coordinator and confirmed by the PFAC participants. They contained written information about what had been informed about, discussed and decided during the PFAC meetings. The meeting minutes were used as a complementary source to the interviews, and the same analytical framework was used. All eleven meeting minutes presented at the web page were analysed. This was for all PFAC meetings between the time period from March 2018 to September 2020.

### Analysis framework

Based on ideas about deliberation, Abelson et al. [26] have described four general principles that can be used to guide the design and evaluation of collective involvement processes in the healthcare sector: (i) representation; (ii) the structure of the process or procedures; (iii) the information used in the process and (iv) the outcomes and decisions arising from the process [26]. Table 2 summarises an adaptation of evaluation criteria linked to the four principles as presented by Abelson et al. This framework was used as a structured analysis matrix and the interviews and meeting minutes were thus analysed through deductive content analysis [27].

**Table 2:** Framework to design and evaluate collective involvement processes in the healthcare sector

Representation	Information	Process and procedures	Outcomes and decisions
Legitimacy and fairness of the selection process: How are representatives chosen? Is there a representative sample? (participant characteristics such as geographical location, demography, disease group)	Decisions regarding what and how information is selected, presented and interpreted	Degree of participant control/input into agenda setting Degree of participant control/input into establishing rules Degree of participation in decision-making Level of participation in the organization: Who is listening and ultimately responding to the participants? Time for deliberation Respect and concern for others’ opinions	Is input incorporated into decisions? Are participants satisfied? Does it improve or change policy-making or service provision?

The framework is an adaption of Abelson et al., 2003 [26].

## Results

### Representation

Most of the interviewed participants were patients or former patients and they had joined the PFAC between one and eight years ago. A large majority of the participants were women. Young people and people born in another country were poorly represented. Some of the PFAC participants were recruited by the RCC as members of local cancer patient organizations, while others were recruited by physicians or friends or without belonging to an organization. A few mentioned that they hesitated before taking on the task, questioning, for instance, what they could contribute and described being encouraged and reassured by RCC representatives. However, most of the participants were, or had been, engaged in other civil society organizations, such as the Red Cross, or sports associations. The meeting minutes show that some changes had been made during the last couple of years to improve representation in the PFAC. The PFAC did, for example, change their assignment description to include two participants from each of the regions attached to the RCC to increase its geographical representation (minute 061218).

The participants seemed to agree that the PFAC should represent cancer patients and their next of kin in a broad sense. For example, one participant said: ‘*You cannot represent yourself when you sit there, because then you do not do much good*’ (Interview #1). A few participants mentioned that although they were recruited based on their experience of a specific type of cancer, they had realized at the PFAC meetings that cancer care needs are similar between different types of cancer patients, and that the PFAC should work for all of them. Some of the participants, however, found this broad perspective difficult and mentioned that since they were recruited from an organization focusing on a specific type of cancer, they had a hard time leaving that specific perspective behind. In line with this, one of the participants mentioned that (s)he sometimes thought that some participants were not over their own illness, and that it affected their ability to focus on the situation of cancer patients more broadly.

Among those participants that were recruited from a local cancer patient organization, the majority tried to inform the local organization about the PFAC’s work, although some mentioned that their local organization was not very interested. One of the participants described that (s)he sent the organization the PFAC meeting protocols: *‘…and then nothing happens, there is no more feedback, or discussions about how to do with this or that. They say thank you and that is that*’ (Interview #8). In two cases, the participants mentioned that they routinely asked their local organization before the PFAC meetings if it had any input to the meeting agenda. In general, the PFAC members thought that it would be an improvement of the PFACs activities if local cancer organizations were more involved.

### Information

The participants mentioned that a large part of the PFAC meetings consisted of information from the RCC, and the meeting minutes confirm that almost all points on the agenda began with some sort of information from the RCC manager or the RCC coordinator. Some participants thought that too much time was dedicated to information, while others thought it was necessary to invigorate and provide structure for discussion. Several of the participants mentioned that that the RCC representatives, who work with the issues on a daily basis, had up-to-date information they wanted to hear. Referring to the RCC manager, one of the participants said: ‘*He is so up-to-date, and we need a lot of information from him, for him to describe how things are now. That is a prerequisite for understanding how it is*’ (Interview # 4). One such example of information was when the manager had informed about how the ongoing pandemic had affected cancer care in their geographical area.

### Process

Concerning the meeting agenda, the participants differed in their views on how it was established. Someone believed that most of the issues on the agenda came from PFAC participants, while others held the view that most were decided by the RCC. Yet a few others said it was a joint effort by the PFAC chairperson and the RCC to establish the agenda and that anyone could make suggestions for what to bring up. For example, one of the participants said: ‘*If I had a suggestion and presented it, and they liked it, it would become part of the meeting agenda’* (Interview #2). Some of the participants mentioned that they had initiated issues to discuss at the meetings, while some had not, sometimes because they felt uncertain in their role.

The participants seemed to agree that, in principle, all had the same opportunity to make themselves heard, even though some were more dominant. Nevertheless, one of the participants brought up that they had to be cautious since some participants may not be as vocal as others, in some cases, because of their illness. A few participants explained that it was a good strategy to hold discussions in smaller groups, as they used to do during the meetings, which helped some to speak up. Most participants mentioned that they agreed with each other to a high extent since everyone wanted to achieve improvements in cancer care and since their illness had given the participants the ability to listen to the experiences of others. However, several of the participants also brought up that the PFAC did not often have the type of discussions that can result in disagreements. One of the participants said: ‘*There are not very many discussions, not as it should be (…) it has not been anything we could disagree on*’ (Interview #8).

Furthermore, it was not clear among the participants what it meant that the PFAC should have an advisory and supportive function vis-a-vis the RCC board. Someone mentioned that the PFAC did not come up with any suggestions that could support the RCC board, while another mentioned that the PFAC should be used more as a referral group and obtain different questions from the board on which to express their views. However, if the advisory role was interpreted more loosely, one of the participants reasoned as follows: *‘…they listen to what we say, of course ... and then we are advisory in a way, right?*’ (Interview #6). It was repeatedly mentioned that the RCC manager and the coordinator partaking in the meetings, who were described as perceptive and professional, listened to the PFAC participants, ‘took things further’ and did not dismiss any ideas, not even very difficult ones. One participant explained: ‘*It is often they say, ‘we will take this to the board, we will present it there’* ‘(Interview #6). This was confirmed by the meeting minutes illustrating that the RCC representatives took suggestions and questions from the participants to the board. The RCC representatives also gave feedback to the PFAC on the process and explained if and how they would continue to work with the suggestions (minutes 140219; 030619; 110520).

Most of the participants were satisfied with the advisory role of the PFAC chairperson in the RCC board. The meeting minutes show that the inclusion of the PFAC chairperson in the board resulted from demands made by the PFAC (minute 060318). One of the participants said that (s)he thought the important thing was that the PFAC had a representative on the board and that someone making decisions was listening. (S)he also said that ‘*the further up in the organization we have representatives, the more likely it is that we get through something we suggest’* (Interview #2). Many of the participants mentioned that they saw the PFACs representation on the board as way to ‘take another step’ or ‘move up a level’; however, they recognized that it requires quite a lot being the representative on the board among all professionals. Notwithstanding, a few participants meant that even if questions were brought up to the board, it did not guarantee any influence on the board’s decisions. One participant exemplified *‘… we can just initiate issues and hope that they [the board] discuss it, and then; well, if they think it is an important issue, they will hopefully pursue it further*’ (Interview # 7). By pursuing it further, the respondents meant that the RCC should take it to the relevant national authorities or decision-makers within the regions.

### Outcomes

The participants had different views on the PFAC’s actual input on the work carried out by the RCC and the issues handled by the RCC board. Some of the participants did not know what input the PFAC had, while some believed it had considerable influence because quite a few of the issues addressed by the board had been brought up by the PFAC. As a rule, the participants thought that the RCC representatives were good at listening to the PFAC participants and that this in itself signalled influence on the RCC’s work. Based on the meeting minutes, the RCC representatives often asked for the PFAC’s perspectives in their work, and the RCC also sought counsel from the PFAC when, for example, developing a new regional cancer plan (minute 030918).

When it came to improvements in cancer care, the participants emphasised that the RCC did a great job, but some were unsure about the PFACs contribution. Most of the participants could not share any specific examples of actual outcomes that the PFAC had had on cancer care. Yet some could, and one example was contact nurses, which was described as an issue pursued intensely by the PFAC. Another example was digital care plans, which a participant said was originally discussed in the PFAC. These plans were described as important for all cancer patients: ‘*…it is an important documentation that the patient can return to when they get home, to remember what the doctor said during the meeting*’ (Interview #6). However, nearly all participants considered the PFAC together with the RCC to be a good forum for providing information to cancer patients and their next of kin, e.g. by producing pamphlets with information concerning issues as involvement and rehabilitation but also by providing resources for meeting points for cancer-specific or local groups. A few participants thought that the PFAC had a lot of relevant information to share but had a problem reaching out and had discussed, for example, how to get information onto the hospitals’ ‘rolling tv screens’.

In several ways, the participants expressed that a lack of knowledge about the healthcare system’s organization was a hurdle to achieving change, although they had taken part in an introductory program for PFAC participants. One gave an example of when the lack of knowledge stopped a PFAC activity. The PFAC had come up with an idea – to place small cards in the waiting areas at hospitals reminding women to ask for ‘my care plan’ – but were told by the RCC coordinator that it could not be done in that way. This was confirmed in the meeting minutes. Another example is that the PFAC wanted to discuss the possibilities of working with smoking cessation in general but learned from the RCC manager that the RCC should mainly focus on secondary prevention for patients that already had received a cancer diagnosis (minute 300518).

Furthermore, one participant explicitly argued that the PFAC was acting on a ‘strange level’ in between the regional and national levels, which in his/her opinion made it harder to exercise influence. (S)he believed that the local cancer councils in the regions were a more direct way to change cancer care. Another participant mentioned that changes at local hospitals had been made quite a few times after criticism voiced by PFAC members in the local cancer councils, although it depended on how open the hospital management was to changes. A few participants also stressed that whether or not the PFAC achieved changes depended to a high extent on the people working at the hospitals, if they listened and changed. Some participants also mentioned that their experiences as cancer patients sometimes came to use in an even better way in the RCC care process groups. One of the participants expressed it like this: ‘*…there they are more interested in me and my experiences and of the care I received and how I felt, and why I felt that way’* (Interview #8).

Overall, despite some hesitation about outcomes, the participants said they were quite satisfied with the effectiveness of the PFAC on improving cancer care. Several mentioned that they would have given a lower rating before, but that many improvements had been made in recent years. Furthermore, the participants reported a high degree of personal satisfaction from being in the PFAC, e.g. being part of a pleasant group and becoming energized. Several expressed that it felt good being able to contribute and help people who were in a difficult situation. One participant expressed it like this: ‘*I use to say that if it helps one person, I am happy. And hopefully I have helped more than that by being in this group*’ (Interview #7). A few also stressed that they had become more knowledgeable about cancer.

## Discussion

### Representation

In terms of representation, which is a recurring theme in studies of PPI [28, 29], the findings suggest that the PFAC participants were recruited with the aim of achieving so-called descriptive representation, where the representative is supposed to have characteristics in common with those represented (in contrast to formal, symbolic or substantive representation) [6]. The descriptive representation was foremost geographical, but there was also a spread in the types of cancer that the participants had or had had. Although most participants tried to take on the perspective of cancer patients in general, it was unclear whether participants recruited from local cancer patient organizations represented their organization or not, pointing to some uncertainty about role, expectations and responsibilities. It was difficult for some to transition from being a member of a local cancer patient organization to being a PFAC participant with a broader perspective. Since several of the participants mentioned that they wished the local organizations would engage more, or actively sought the input from their local organizations, it appears as if the PFAC participants preferred a delegate model of representation, where representatives act on the stated preferences of those represented, in contrast to a so-called trustee model in which representatives follow their own thoughts regarding the right action [6].

### Information and process

Concerning information, the findings suggest that the RCC had a large part in setting the meeting agenda (an enabler/barrier mentioned by, for example, Bombard et al. [30]) and that an important aspect of the PFAC meetings was information sharing by the RCC representatives, which many participants saw as necessary for relevant discussions. The findings related to the process suggest that power seemed to be distributed evenly between the participants, but it was mentioned that the participants’ personality played a major role in how much space they took and that those with greater self-confidence and physical ability spoke more [31]. Research on preferred characteristics of PFAC members has suggested that they should have good communication skills, be ‘good listeners’ and have a ‘passion for helping’, and those aspects were mentioned in the interviews as preventing conflicts from taking place and as a driver of participation.

Furthermore, the PFAC participants emphasised that the RCC representatives were crucial for the functioning of the PFAC, and in a similar way, it has been pointed out in previous research that support is necessary both for practicalities such as meeting rooms and meals and to keep the PFAC on track by providing an agenda, keeping minutes and providing guidance to keep the PFAC on topic during meetings [6, 32]. In this case, the RCC representatives organized and prepared the meetings and provided resources. Importantly, it has been noted that this may compromise the independence of a PFAC [6]. In a similar way, it has been suggested that patient organizations may have a dependent position *vis-à-vis* providers or insurers, which results in a risk that they are put to instrumental use [8], i.e. that the inclusion in policy processes or decision-making may be used as a way to add legitimacy to decision-making bodies. However, this was not reflected in the interviews; rather the PFAC participants saw the RCC representatives as allies against the regions or hospitals when cancer care did not function properly and the RCC representatives could thus be thought of as external facilitators [30] in the relation between patients/next of kin and cancer care services and policy-makers.

### Outcomes

The literature points out that outcome evaluation is difficult. It is, for example, difficult to evaluate how and when influence occurs and to establish the right end-point or timeframe for effects [26]. Some outcomes may be proximal, while others may be distal, and institutional or societal effects can take years to appear. In line with this, our findings show that the PFAC participants thought it was difficult to know whether the council’s activities had led to any changes to cancer care or policy, but were very positive towards the RCC’s work to improve cancer care. The PFAC participants generally thought it was a positive outcome if the issue they had raised ‘was taken further to a higher level’ by the RCC and were all enthusiastic over the opportunity to make their voices heard in this type of forum, which at least gave a potential for influence because someone with power over cancer care listened. This means that the PFAC responded to the moral argument for PPI: i.e. that those affected by a health service should have a say in what is done and how it is done. However, it is uncertain to what extent it responded to the consequentialist argument: i.e. whether it improved the quality or efficiency of cancer care [2]. By that, the impact of PFACs is still uncertain regarding healthcare decisions at the population level [6, 14, 15]. Future studies should focus on how to measure the outcomes of PFACs in organizational change or policy, what Dukhanin et al. [28] refer to as internal outcomes of PPI on the services provided by an organization or system and on the organization or system itself. This type of organizational impact is more difficult to measure than impact on the participants, such as increased knowledge, skills or satisfaction [20, 33].

### Challenges: system knowledge and clarity of role and rationale

Earlier research has suggested that participants should be familiar with the healthcare system within which the PFAC is located [6], and this was mentioned as an obstacle in the interviews. Although the participants thought it was a positive outcome that an issue ‘was taken further to a higher level’ by the RCC, it was often unclear to them which level was referred to and what the decision-making capacity was at that level. The PFAC participants were generally familiar with how local cancer care provision worked but were less familiar with how the system works at the managerial level (meso- and macro-levels), e.g. where different types of decisions are being made and which channels should be used to push for different types of changes. Furthermore, in previous research, it has been put forward that PFACs need to be located within the organizational structure in a way that it could make real decisions and have a meaningful impact [6]. In the interviews, it was questioned if this was the case with the PFACs attached to the RCCs, which work in between the national and regional governing levels – again pointing to uncertainties about the PFAC’s role and rationale. Some participants saw more directly how the patient experience could be used in the local cancer councils in the regions or in the care process groups, which include responsible healthcare staff, and are closer to the operational level in the healthcare organization. This indicates that it is more challenging for patients and their next of kin to partake in involvement activities at the organizational and policy levels, making it particularly important to create a receptive context where participants have sufficient guidance and support [30]. This may include offering participants relevant education and training [24, 29] and trained facilitators that have a comprehensive knowledge about how the healthcare system works and who can support the participants in navigating the managerial level of the healthcare system. This may require additional training of the facilitators as well [34].

Institutional commitment is often described as a facilitator/barrier to successful involvement [30, 31] and the RCCs have established a multifaceted system for involving patients and next of kin. It can, however, be discussed to what extent the current PFAC mandate incorporates the potential for influencing policy and service improvement. According to its mandate, the PFAC is to perform an advisory and supportive function *vis-à-vis* the RCC board, but some of the representatives did not fully understand what that entailed and what was expected of them, thus being a barrier to action. Furthermore, the potential for influence is dependent on how the participatory activity is set up [31]. Often, the level of control over decision-making in PPI is described as stretching from *consultation* (an opportunity to express views) to *partnership* (shared planning and decision-making responsibilities) to *lay control* (autonomous decision-making authority) [35], with a first level sometimes being added: *information* (being informed). Because the chairperson only has the right to speak in the RCC board and not take part in decisions, its role is by definition consultative. As such there is no guarantee that the PFAC will have an impact on the development of cancer care or policy, only that the participants get an opportunity to express their views (which they wished they were asked to do in a more systematic way). Thus, if the PFAC is to be guaranteed influence within the RCC, a change of the mandate is necessary, e.g. to give the chairperson authority to take part in decisions in the RCC board. However, this would raise the demands for more formal accountability procedures and a more formal process for recruiting participants to reach the desired type of representation, which has previously been described as sensitive and time-consuming [31]. Overall, our results point to the importance of discussing and clarifying roles, expectations and rationales linked to PPI activities in order to optimize their effectiveness.

### Limitations

Lastly, there are some limitations to this study. One is that the study only investigated one of six RCC PFACs, which may work differently. Also, the sample of participants is relatively small, although this was remedied in part by validating the interviews against the meeting minutes from the PFAC. Another limitation is that the study did not include observations of the meetings, which would have contributed with a deeper understanding of the dynamics in the PFAC. Furthermore, it is not possible to know whether the PFAC participants who refrained from partaking in the study were more negative towards the PFAC or if they differed regarding some other aspect. In addition, there may be a difference between perceived and actual outcomes, which may in turn be linked to recall problems. Also, outcomes may appear only after some time and may thus not be possible for the participants to detect at a particular time. Since there was variation in how long the PFAC participants had partaken in the council, they may also have had different opportunities to assess the PFAC's outcomes.

## Conclusion

The effectiveness of PFACs is uncertain, and the investigation of a PFAC for cancer care operating at the managerial level in a healthcare system shows that, although the representativeness and the participatory processes had been improved, there was uncertainty about the role and expectations of the participants and of the PFAC’s outcomes. It was difficult for the PFAC participants to know whether their efforts had led to any improvements to cancer care or policy. The participants were, however, satisfied with being listened to but felt they needed to understand the meso- and macro level functioning of the healthcare system better to improve their work. The study thus suggests that it is more difficult for patients and family members to participate at the managerial level in healthcare organizations compared with the operational level with which they are usually more familiar, although it is at these levels strategic decisions about priorities in cancer care and service configurations are made. Thus, there is a particular need for guidance and support for PPI to work successfully at this level, which may include relevant education and training in system knowledge.

## Data Availability

Data sharing is not applicable to this article as no datasets were generated or analysed during the current study.

## Abbreviations

ECPC: The European Cancer Patient Coalition
LCC: Local cancer council
PFAC: Patient and Family Advisory Councils
PPI: Patient and public involvement
RCC: Regional cancer centre
